# Phenolic Profiles, Antioxidant and Anti-Inflammatory Activities of Hydrodistillation Wastewaters from Five Lamiaceae Species

**DOI:** 10.3390/molecules27217427

**Published:** 2022-11-01

**Authors:** Edoardo Napoli, Giuseppe Ruberto, Alessandra Carrubba, Mauro Sarno, Claudia Muscarà, Antonio Speciale, Mariateresa Cristani, Francesco Cimino, Antonella Saija

**Affiliations:** 1 Institute of Biomolecular Chemistry, National Research Council (ICB-CNR), Via P. Gaifami 18, 95126 Catania, Italy; 2 Department of Agricultural, Food and Forest Sciences, University of Palermo, Viale delle Scienze, Build 4, Entr. L, 90128 Palermo, Italy; 3 Department of Chemical, Biological, Pharmaceutical and Environmental Sciences, University of Messina, Viale Ferdinando Stagno D’Alcontres 31, 98166 Messina, Italy

**Keywords:** distillation wastes, phenolic profile, rosmarinic acid, intestinal inflammation, anti-inflammatory, radical scavenging, antioxidant

## Abstract

Distillation is the most widely used method to obtain an essential oil from plant material. The biomass used in the process is returned as a solid residue together with variable amounts of water rich in water-soluble compounds, which currently are not addressed to any further application. The scope of this work was to evaluate the phytochemical composition of wastewaters coming from hydrodistillation (DWWs) of five aromatic plants belonging to the *Lamiaceae* family, and to assess their in vitro antioxidant and anti-inflammatory activities. The phenolic profiles of the DWWs were determined by HPLC-DAD and HPLC-ESI/MS. Free radical scavenging ability, oxygen radical antioxidant capacity and superoxide dismutase mimetic activity of the samples under study were measured. Moreover, to investigate the anti-inflammatory activity of the DWWs, an in vitro experimental model of intestinal inflammation was used. The DWW samples’ phytochemical analysis allowed the identification of 37 phenolic compounds, all exhibiting good antioxidant and anti-inflammatory activity. Our study contributes to the knowledge on the polyphenolic composition of the DWWs of five aromatic plants of the Lamiaceae family. The results highlight the presence of compounds with proven biological activity, and therefore of great interest in the pharmaceutical and nutraceutical fields.

## 1. Introduction

The cultivation of medicinal and aromatic plants (MAPs) has recently been receiving a worldwide re-evaluation. In former years, MAPs were considered as typical productions of small-scale agricultural systems, and mostly relegated to marginal areas. Nowadays, MAPs are increasingly intended as crops capable of providing a significant profit margin, despite the limited initial investment. Most of the production is processed on site and intended for food use as spices, without further processing steps [1]. Through a distillation process, some MAPs are able to produce essential oils (EO), widely used in aromatherapy, phytotherapy, in the cosmetic and in food industries [2,3,4]. Additionally, some of them, such as oregano, rosemary, sage, thyme and, more recently, lavender, are attracting the attention of producers for the potential utility of the by-products coming from their production processes [5,6].

Distillation is the most widely used method to obtain an EO from plant material, and thus far, it is the only allowed method by international regulations to obtain a product termed an essential oil [7,8]. Water distillation (or hydrodistillation) and steam distillation are the most common procedures. The former occurs by submerging the chopped plant material into boiling water, and the latter uses a steam flow passing through the raw material, generated by an external source, or by boiling water underneath [9,10]. In both cases, the biomass used in the process undergoes a thermal treatment (by direct contact with the water brought to boiling or by the passage of water vapor produced in special boilers) and is returned as an exhausted residue (without the volatile parts that constitute the essential oil), together with variable amounts (depending on the type of distillation used and the type of plant) of water rich in water-soluble compounds. The residual water collected at the end of the cycle in the current steam plants can increase from a minimum of 5% of that initially introduced into the system in the case of large industrial plants with a separate boiler to 70–80% in the case of smaller systems that use the closed boiler with water recirculation, and it can reach up to 90–95% in the cases of small-bench hydrodistillation systems. The solid residue (weighing about 5% more than the vegetable matrix subjected to distillation, due to wetting during the process) is currently destined either to produce thermal energy by combustion, or to composting. The residual water, instead, is currently not addressed to any further applications and is therefore discharged. The industrial production of EO takes place through highly demanding processes (transport of the plant material, heating of the boilers to produce steam). Although the market for the final products (EO and/or hydrolates) is growing, issues are beginning to emerge about the economic/environmental management of the considerable amount of exhausted biomass unsuitable for the production of energy through bio-digestion, as well as about the eco-sustainability of the whole process. Consequently, the need arises to find new ways of exploiting distillation waste, which, by transforming it from “waste” to “secondary raw material”, can totally or partially reduce the economic and ecological issues from EO production, according to the principles of bio-refinery. The wastewater obtained after the MAPs’ distillation process can be likened to the product of a hot aqueous extraction from a plant matrix, therefore rich in all the water-soluble and thermostable metabolites present in the starting matrix, and in any artifacts due to the process. It is a “green” product since no solvent is used in the process. The distillation water is therefore rich in biologically active substances, polyphenols in particular, as already reported in the literature [11,12]. These residues can be considered phytocomplexes, which represent the basis of modern nutraceuticals. Like multi-target ligands, phytocomplex exploits the “herbal shotgun” effect, where multiple constituents interact with different targets, in contrast with the “silver bullet” effect, that refers to the action of a single substance on a single target [13]. However, the possibility to exploit the residual wastewaters has not yet been explored, mostly because of the lack of specific studies on their composition and properties.

The scope of our work has been to evaluate the phytochemical composition of wastewaters coming from hydrodistillation of five MAPs belonging to the *Labiatae* family (*Rosmarinus officinalis* L., *Origanum vulgare* L., *Origanum majorana* L., *Salvia officinalis* L. and *Thymus vulgaris* L.), as well as to assess their in vitro antioxidant and anti-inflammatory activities, with the aim to discover useful information for their exploitation in the pharmaceutical and nutraceutical fields.

## 2. Results

### 2.1. HPLC Polyphenolic Profiles of Wastewaters

**Figure 1:**
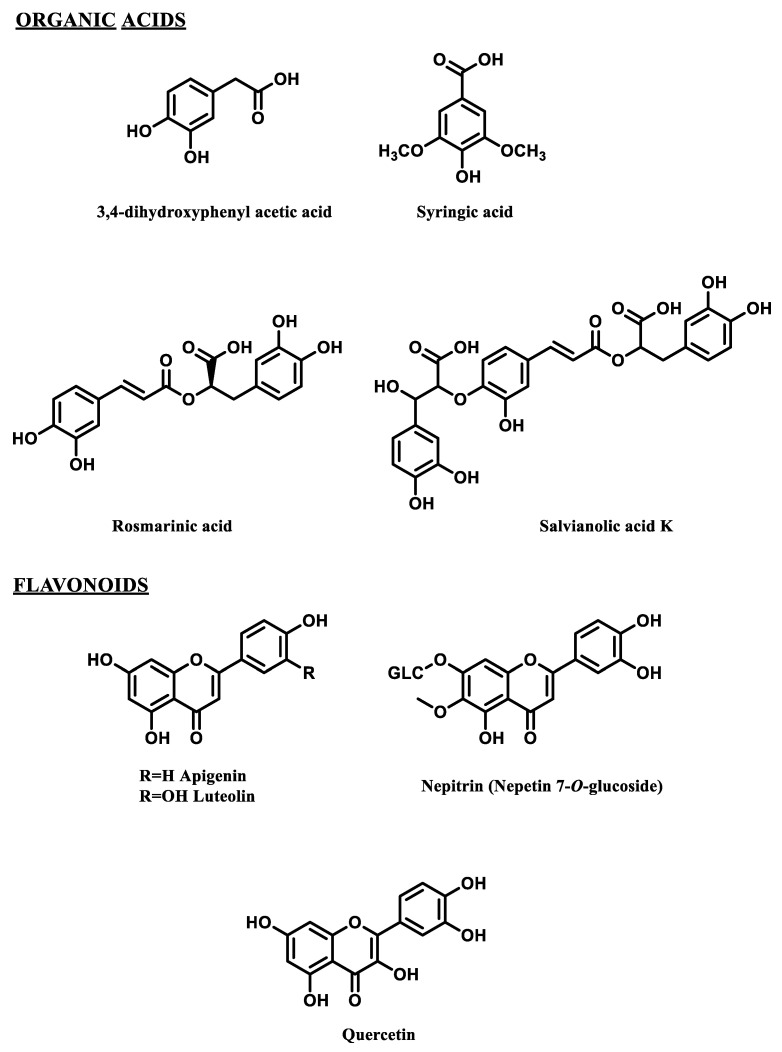
Selected polyphenols detected in the wastewaters from hydrodistillation of 5 aromatic species.

Phytochemical evaluation of the five aromatic species was carried out through the HPLC identification of the main polyphenolic compounds (Figure 1).

Table 1 lists the quantitative data of the 37 tentatively identified polyphenols, along with the results of the ANOVA and Tukey’s test performed.

The highest amounts of active compounds (175.26 and 161.28 mg g^−1^, respectively) were found in the wastes from sage (*Salvia officinalis* L.) and common thyme (*Thymus vulgaris* L.). The other three species afforded lower amounts, from 134.24 mg g^−1^ (*Rosmarinus officinalis*) to 129.75 mg g^−1^ (*Origanum majorana*) and 124.86 mg g^−1^ (*Origanum vulgare*).

Striking differences among species were also found in the wastewater composition. In all species, organic acid derivatives were the most abundant fraction (from 123.38 mg g^−1^ in *S. officinalis*, corresponding to 70.5% of the total retrieved compounds, to 88.22 mg g^−1^ in *O. majorana*, i.e., 67.5% of the total). Although 19 compounds belonging to this chemical family were identified, each species was composed by a limited number of them (never more than 6–7, with the first 3 usually able to reach about 90% of total organic acids identified). Within this group of compounds, rosmarinic acid was always the most abundant, reaching 70.41 mg g^−1^ (52.1% of the total compounds) in *R. officinalis*, 49.35 mg g^−1^ (39.5%) in *O. vulgare*, and 54.78 mg g^−1^ (30.7%) in *S. officinalis*. Only two other organic acid derivatives were present in all species, although at different amounts: dihydroxyphenylacetic acid, from 2.79 mg g^−1^ in *T. vulgaris* to 20.39 mg g^−1^ in *O. vulgare*, and ferulic acid derivative, from 2.23 mg g^−1^ in *O. majorana* to 6.04 mg g^−1^ in *T. vulgaris*. Dihydroxyphenylacetic acid was the second most abundant organic acid derivative in *O. vulgare* (16.3% of total compounds) and *R. officinalis* (12.8%), whereas salvianolic acid K was second in *S. officinalis* (35.9 mg g^−1^, 20.1% of the total compounds) and *T. vulgaris* (18.4 mg g^−1^, 11.4% of the total compounds). In *O. majorana*, the second most abundant compound was a caffeic acid tetramer B (15.46 mg g^−1^, 11.8%). Noticeably, *T. vulgaris* also contained appreciable amounts of a caffeic acid trimer 2 (13.24 mg g^−1^, 8.2%).

Flavonoid derivatives (Table 1) were found in lower amounts (51.88 mg g^−1^ in *S. officinalis*, 42.91 mg g^−1^ in *T. vulgaris*, 41.90 mg g^−1^ *R. officinalis*, 41.53 mg g^−1^ in *O. majorana* and 30.77 mg g^−1^ in *O. vulgare*) and they were mostly (totally, in *O. vulgare*) represented by flavones, that formed 64% to 94% of all flavonoid derivatives, and in lower amounts by flavonols.

As previously stated for the group of organic acids, in each species, flavonoids were also present with 5–6 different compounds, the first of which was dominant on all the other flavonoids detected. In four out of five species, this predominant flavonoid was luteolin 7 *O*-glucuronide, the only flavonoid detected in all species. It was the most abundant flavonoid in *S. officinalis* (28.49 mg g^−1^, 16.0% of the total compounds), *T. vulgaris* (26.24 mg g^−1^, 16.3%), *O. majorana* (26.07 mg g^−1^, 20.0%) and *O. vulgare* (8.97 mg g^−1^, 7.2%). Lower amounts were found in *R. officinalis* (3.28 mg g^−1^, 2.4% of the total identified compounds), where otherwise significant amounts were detected of luteolin 3 *O*-glucuronide (15.64 mg g^−1^, 11.6%), which took the place of the “dominant” flavonoid. Luteolin derivative (6.55 mg g^−1^) was retrieved only in *O. vulgare*, where four derivatives of apigenin were found as well (apigenin derivative A, apigenin derivative B, apigenin 7 *O*-glucoside and methyl apigenin *O*-glucuronide). Nepitrin was detected only in *S. officinalis* (10.12 mg g^−1^), and an apigenin glucuronide (10.51 mg g^−1^) in *O. majorana*.

Flavonols were most abundant in *T. vulgaris*, where they were retrieved in three forms (quercetin 3 *O*-glucoside, quercetin derivative and quercetin 3 *O*-rhamnoside), and in *R. officinalis*, where the only flavonol present was quercetin 3-*O*-glucuronide.

### 2.2. Evaluation of the Antioxidant Properties

The antioxidant/radical scavenger activities of the five wastewaters submitted to this study are listed in Table 2.

The observed activities did not appear to be clearly correlated to the content of total polyphenols or to that of the individual types of antioxidants; for example, *O. majorana* and *R. officinalis*, despite that their polyphenol content was similar to that of *O. vulgare*, showed a clearly lower antioxidant activity in all tests performed. However, it is known that the reaction environment influences the test results, which are therefore related more to the chemical-physical characteristics of the individual compounds present in these matrices than to the total polyphenol content. On the other hand, the wastewaters examined in our study are very complex matrices containing a number of bioactive components which can all contribute to the whole effect, also behaving as pro-oxidants [14,15] or through interactions (antagonism or synergism) among them [16]. It should be noted that *O. vulgare*, *T. vulgaris* and *S. officinalis* are much more effective than *R. officinalis* and *O. majorana* both in the ORAC test and in the SOD assay, both useful for determining the effectiveness against biologically relevant radicals. Among these assays, ORAC is a hydrogen atom transfer (HAT) reaction-based assay, while the DPPH assay is usually classified as an electron transfer (ET) reaction, although a mixed mechanism has also been hypothesized (ET/HAT) [17,18,19]. The ORAC assay is the only method that takes free radical action to completion and uses an AUC technique for quantitation. However, ORAC is not suitable for measuring lipid-soluble antioxidants, while DPPH• is soluble only in organic solvents; furthermore, in the SOD assay (where the mechanism is not clearly defined), superoxide radicals are generated in hydrophilic medium. As for flavonoids found in these waters, they are always present as glycosides or glucuronides. Flavonoid glucuronides and glycosides are more hydrophilic, but usually their biological activity is lower than that of corresponding aglycones [20,21,22]. *O. vulgare*, which definitely lacks flavonols, is one of the most active products in all assays (particularly ORAC and SOD assays), while *O. majorana* is very rich in flavones but has a poor response to antioxidant assays. This seems to confirm the almost insignificant contribution of these glycosylated and glucuronidated flavonoids to the antioxidant activity of the waters investigated. For organic acids, it is worth mentioning the presence of caffeic acid trimers (abundant in *T. vulgaris* and, to a lower extent, in *S. officinalis*) and tetramers (abundant in *S. officinalis* and *O. majorana*). Although only few and inconsistent data are reported about the antioxidant power of caffeic acid polymers, they seem to maintain a good antioxidant activity that is influenced by the binding position and substituent presence [23]. Finally, dihydroxyphenylacetic acid, recovered especially in *O. vulgare*, is known for its antioxidant power [24]. In conclusion, the antioxidant activity of these waters may be significantly influenced by the presence of (quantitatively) secondary components, capable of acting against superoxide and peroxyl radicals in an aqueous medium.

### 2.3. Statistical Analysis

**Figure 2:**
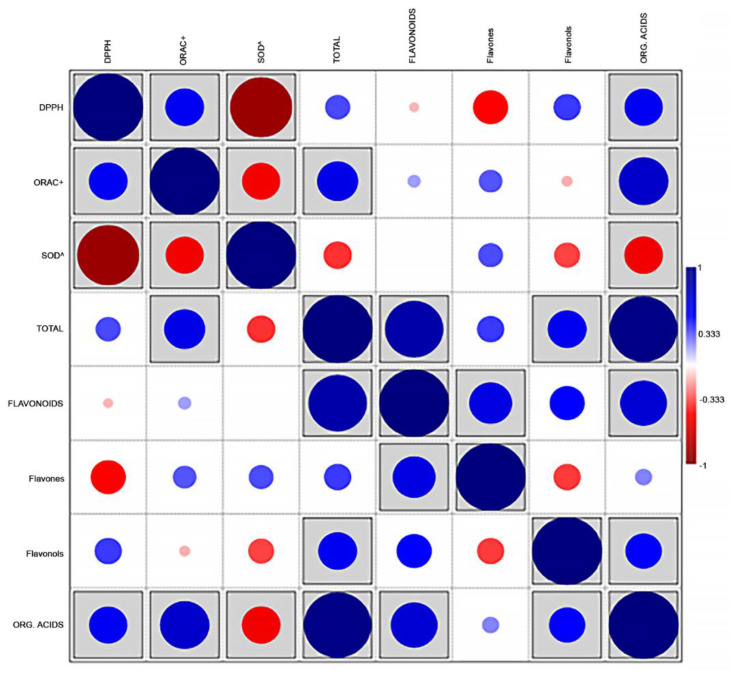
Correlations plot (Pearson’s r) between the main families of compounds and the values of antioxidant activity in 5 aromatic species (*n* = 15). Boxed r values are significant at *p* < 0.05.

Correlation analysis (Figure 2) revealed that the total metabolite content was positively and directly associated (r = 0.967, *p* < 0.001) with the content in organic acids, which represented the majority fraction.

This measurement is also directly correlated to the flavonoid content (r = 0.834, *p* < 0.001) and, to a lesser, although significant, extent, to the flavonols content (r = 0.551, *p* = 0.033). The sum of flavonoids was, expectedly, correlated with their major components, i.e., flavones (r = 0.609, *p* = 0.016), but also to the amounts of organic acids (r = 0.666, *p* = 0.007). Significant associations were detected between the content in organic acids and all measurements of antioxidant activity (r = 0.705, significant at *p* = 0.003; r = 0.543, significant at *p* = 0.036; r = −0.548, significant at *p* = 0.034, with ORAC, DPPH and SOD values, respectively). DPPH and ORAC (expressed as TE μmol/mg dm) were positively associated with each other (r = 0.549, *p* = 0.034), whereas the values of SOD (expressed as SC_50_ mg dm/mL) followed an opposite trend, being inversely correlated with DPPH and ORAC (r = −0.889, *p* < 0.001, and r = −0.541, *p* = 0.037, respectively). Unexpectedly, the flavones content showed a significant inverse correlation with DPPH measurements (r = −0.497, *p* = 0.006).

The PCA allowed a deeper insight of the relations among data. The first two principal components (PC1 and PC2) explain 78.56% of the total variation in analytical data (Table 3) and show clear partitioning of all species along the two axes. *O. majorana* and *S. officinalis* are well-distinguished along the PC1, whereas *O. vulgare* is clearly separated from *O. majorana* and *S. officinalis* along the PC2. However, Table 3 shows that the PC3 must also be considered significant, as the percentage of variance explained when including PC3 accounts for 96.45% of the total variance. The third axis allows separating *R. officinalis* and *O. vulgare*, that based on the first two PCs appeared associated.

The first PC is more associated with the content of organic acids and the total content of active metabolites, whereas the content of flavones, and to a lesser extent, flavonoids, are well-explained by PC2. PC3 is otherwise linked to the content in flavonols.

**Figure 3:**
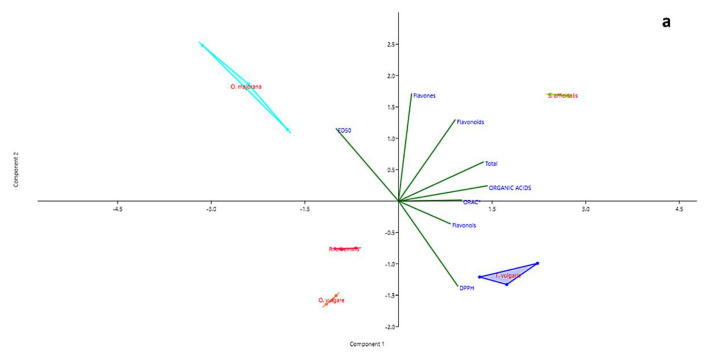

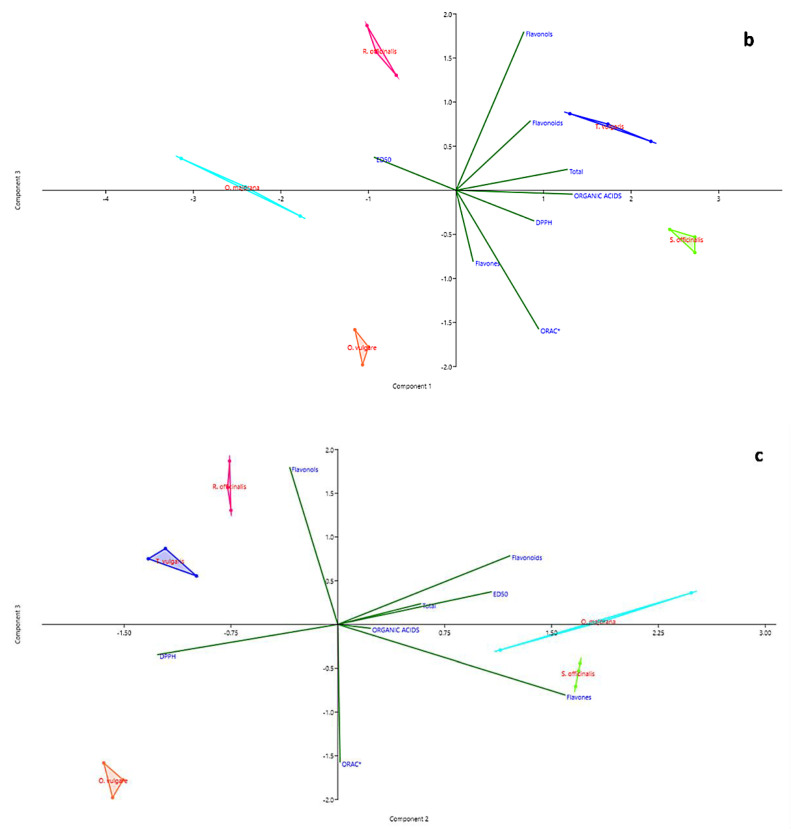
PCA biplots (PC1 and PC2 (**a**), PC1 and PC3 (**b**), PC2 and PC3 (**c**)) for families of chemical compounds and measurements of antioxidant activity in distillation wastes of *Origanum vulgare* L., *Rosmarinus officinalis* L., *Origanum majorana* L., *Thymus vulgaris* L. and *Salvia officinalis* L.

In the plane formed by the first two PCs (Figure 3a), the content in organic acids seems tightly associated to the ORAC values, whereas when PC3 is considered (Figure 3b,c), a closer association appears between organic acids and DPPH values.

**Figure 4:**
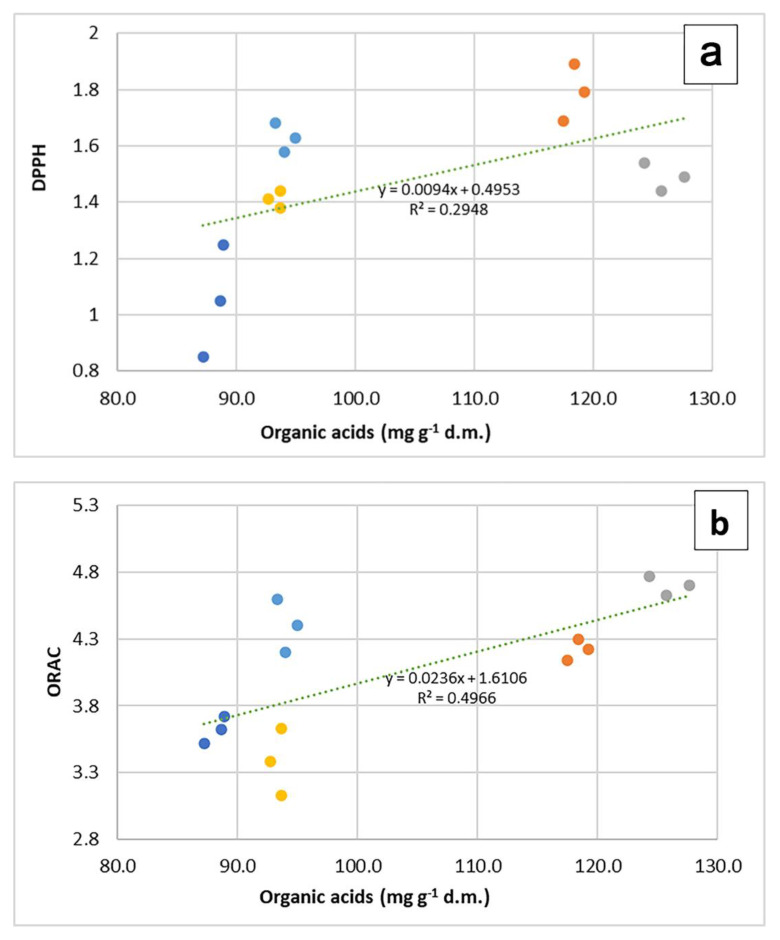

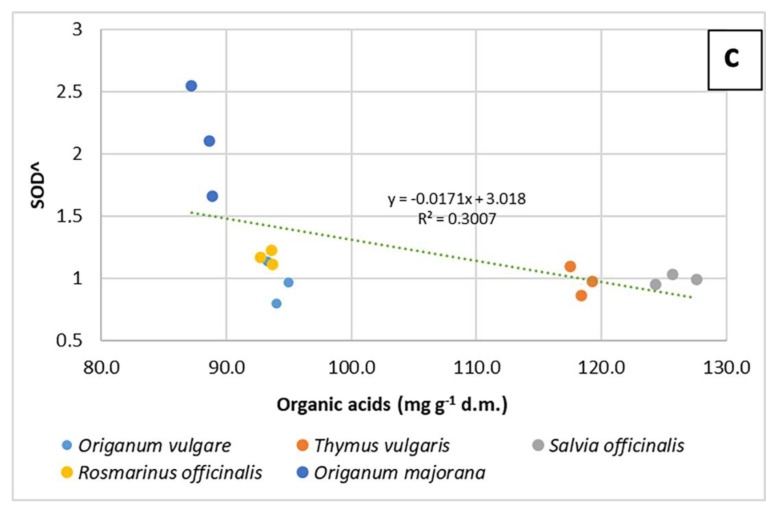
Regression plots of DPPH (**a**), ORAC (**b**) and SOD^ (**c**) values on organic acid content in 5 aromatic species. In each graph, regression lines and equations refer to the pooled data (*n* = 15).

In all three representations, whatever their position, the vectors representing the values of DPPH and ED_50_ always take opposite directions. The regression plots between all three measurements of antioxidant capacity and the total content of organic acid in samples (Figure 4a–c) show how the seemingly good fitting of pooled data, as seen by means of correlation analysis, is however markedly different according to the species. The observation of graphs allows partitioning the five species into two groups, composed by *O. vulgare*, *O. majorana* and *R. officinalis*, dealing with low amounts of organic acids, and *S. officinalis* and *T. vulgaris*, characterized by higher amounts of these compounds.

### 2.4. Anti-Inflammatory Activity in Intestinal Epithelial Cells Exposed to TNF-α

Polyphenols have shown beneficial effects in intestinal diseases, and in particular may be taken into consideration as therapeutic agents in the prevention and treatment of inflammatory bowel diseases (IBDs), acting through multiple mechanisms besides the antioxidant properties [25,26]. Thus, we used an in vitro experimental model of intestinal inflammation consisting of intestinal epithelial Caco-2 cells exposed to the pro-inflammatory cytokine TNF-α to investigate the anti-inflammatory activity of the wastewaters. Exposure to TNF-α induces the transcription of pro-inflammatory genes through the activation of the NF-κB pathway, thus promoting the expression of other inflammatory cytokines, such as interleukins IL-6 and IL-8 [27]. NF-κB is a heterodimer composed of the p50 and p65 subunits and, in unstimulated cells, is sequestered in the cytoplasm by the inhibitor of kB (IkB). Activation of cells with various stimuli initiates a signaling cascade that finally leads to the disruption of the inactive complex and to the nuclear translocation of NF-κB, with the consequent activation of pro-inflammatory genes [28].

To study the effects of the wastewaters on the inflammatory response, the activation of the NF-κB pathway was evaluated by determination of nuclear p65 through Western blotting. Moreover, COX-2 protein levels have also been evaluated. COX-2, in fact, is an inducible enzyme affecting prostaglandin and thromboxane production, regulated by the NF-κB pathway [29], and involved in the pathogenesis of IBDs [30].

**Figure 5:**
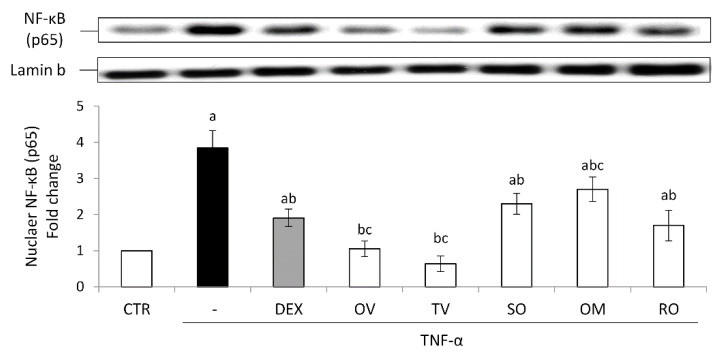
Nuclear NF-κB (p65). The Caco-2 monolayer was pretreated with the extracts for 24 h, and subsequently exposed to 50 ng/mL of TNF-α for 6 h. Cultures treated with the vehicles alone were used as controls (CTR). Dexamethasone (DEX), 10 µM for 1 h, was used as a positive control. Caco-2 nuclear lysates were analyzed by Western blotting, and nuclear localization of the p65 protein was evaluated. NF-κB (p65) intensity values were normalized to the corresponding Lamin-B value. Results are reported as fold change against control and expressed as mean ± SD of three independent experiments. ^a^ *p* < 0.05 vs. CTR; ^b^ *p* < 0.05 vs. TNF-α; ^c^ *p* < 0.05 vs. DEX + TNF-α. OV: *Origanum vulgare* L.; RO: *Rosmarinus officinalis* L.; SO: *Salvia officinalis* L.; OM: *Origanum majorana* L.; TV: *Thymus vulgaris* L.

**Figure 6:**
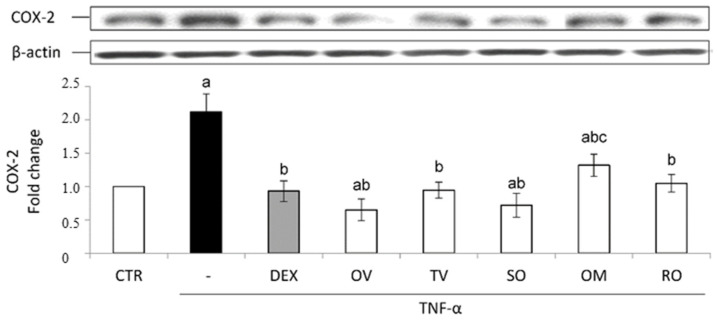
COX-2 protein expression. The Caco-2 monolayer was pretreated with the extracts (50 µg/mL) for 24 h, and subsequently exposed to 50 ng/mL of TNF-α for 6 h. Dexamethasone (DEX), 10 µM for 1 h, was used as a positive control. Cultures treated with the vehicles alone were used as controls (CTR). Caco-2 whole cell lysates were analyzed by Western blotting, and the expression of the COX-2 protein was evaluated. Results are reported as fold change against control and expressed as mean ± SD of three independent experiments. COX-2 intensity values were normalized to the corresponding b-actin value. ^a^ *p* < 0.05 vs. CTR; ^b^ *p* < 0.05 vs. TNF-α; ^c^ *p* < 0.05 vs. DEX + TNF-α. OV: *Origanum vulgare* L.; RO: *Rosmarinus officinalis* L.; SO: *Salvia officinalis* L.; OM: *Origanum majorana* L.; TV: *Thymus vulgaris* L.

All the investigated wastewaters induced a significantly good anti-inflammatory effect. The treatment with TNF-α induced a significant nuclear accumulation of the transcription factor NF-κB and consequently an increased expression of the inducible COX-2. Both these effects were prevented by the treatment with the anti-inflammatory steroid dexamethasone used as a positive control (Figure 5 and Figure 6).

All the wastewaters were able to prevent NF-κB nuclear accumulation induced by TNF-α, showing the following efficacy order: *T. vulgaris* > *O. vulgare* >> *R. officinalis* > *S. officinalis* > *O. majorana*. Interestingly, the effect of *T. vulgaris* and *O. vulgare* was more marked than that induced by dexamethasone (Figure 5).

In accordance with p65 data, as to COX-2 intracellular levels, all the waters produced a protective effect, which was similar to that induced by dexamethasone, except in the case of *O. majorana* (Figure 6).

In this case, the importance of the complexity of the vegetable matrix is also evident, in relation with the efficacy of the less representative compounds and with the effect of the possible interaction among the present compounds. This is especially evident in the results concerning *R. officinalis* wastewater, which, despite having a total phenols content similar to that of *O. vulgare* wastewater, exhibits a significantly lower activity than the other, not only as concerns the antioxidant effect (see data of the ORAC assay) but especially for the anti-inflammatory properties. Similarly, despite their similar total phenolic content, *T. vulgaris* has a stronger anti-inflammatory effect than *S. officinalis*.

The main components found in these waters have been reported in the literature as endowed with anti-inflammatory properties. Rosmarinic acid as well as syringic acid (the last being present only in *O. vulgare* and *T. vulgaris*) can ameliorate intestinal inflammation, as demonstrated in experimental in vivo and in vitro models [31,32,33,34]. Dihydroxyphenylacetic acid was shown to decrease COX-2 expression in human colon LT97 cells [35] and reverse inflammatory responses in human intestinal epithelial cells [36]. Furthermore, luteolin and luteolin glucuronides have anti-inflammatory effects in lipopolysaccharide-treated RAW264.7 cells, although luteolin glucuronides (especially luteolin 7 *O*-glucuronide) have weaker effects than those of luteolin [37].

## 3. Discussion

Polyphenols are among the most studied classes of phytochemicals, due to their ubiquitous distribution in the plant kingdom and to their numerous biological properties, such as antioxidant, antimicrobial, anti-inflammatory and modulatory activity of the cardiovascular system. These findings make the polyphenolic-based phytocomplexes interesting for nutraceuticals and pharmacology. Extracting polyphenols from plants for industrial exploitation puts plant biodiversity at risk and involves the use of solvents and energy, often making the processes ecologically and economically unsustainable. The researchers’ attention therefore turned to agri-food industry waste as an alternative source [38]. The industrial production of essential oils involves, as already mentioned, a considerable waste of primary resources (energy and water), returning a high added-value product (essential oils and hydrolates), to the detriment of large quantities of exhausted vegetable biomass and wastewater which are rich in bioactive metabolites, many of which are polyphenolics. Our work addressed the re-evaluation of these wastes, by promoting their reuse as a source of bioactive metabolites.

The analysis of the polyphenolic component of the distillation wastewater of the five MAPs considered in this study showed that it contains 8–12% of organic acids, of which only 5–7% of rosmarinic acid and up to 20% of dihydroxyphenylacetic acid, which can be considered as its degradation product. Rosmarinic acid is an ester of caffeic acid and 3,4-dihydroxyphenyl lactic acid. In plants, rosmarinic acid acts as a preformed, constitutively accumulated defense compound [39]. Plenty of biological activities have been described for rosmarinic acid: astringent, antioxidative, anti-inflammatory, anti-mutagen, antibacterial and antiviral. Many reports have demonstrated that rosmarinic acid has an important role in treating inflammatory diseases through multiple mechanisms and exerts anti-inflammatory effects to treat various diseases [40]. Rosmarinic acid demonstrated a hepato-protective effect against lipopolysaccharide- and d-galactosamine-sensitized mice [41]. Rosmarinic acid appears to be an effective candidate for inclusion in functional foods and pharmaceutical plant-based products, as demonstrated in several studies conducted on feedstock animals. The benefits of dietary rosmarinic acid supplementation as a phytogenic additive are promising since it acts as a powerful antioxidant and growth-promoting agent [42]. Given the current evidence, it may be included in the daily diet in the treatment of several diseases, with a very low cytotoxicity risk [43]. In the list of biologically active compounds identified in the distillation wastewaters, there are also the derivatives of the phenolic acids caffeic and ferulic acid. Caffeic acid and its derivatives have been used up to now for their medicinal properties. Caffeic acid possesses various biological and pharmacological activities, including antioxidant, anti-inflammatory and neuroprotective effects. All these activities are mediated via repression and inhibition of transcription and growth factors [44]. Recently, the anti-tumor activity of caffeic acid has also attracted much attention due to its capability to induce apoptosis in cancer cells via enhancing reactive oxygen species (ROS) levels and impairing mitochondrial function. Molecular pathways involved in cancer progression are affected by caffeic acid and its derivatives. Additionally, caffeic acid seems to suppress metastasis by inhibiting the epithelial-to-mesenchymal transition mechanism [45]. Some salvianolic acids derivatives have been detected in our samples, with a remarkable presence of salvianolic acid K in thyme and sage wastewaters. Salvianolic acids constitute a valuable class of natural compounds with potential for the treatment of fibrosis diseases and cancer, and it is worth mentioning that the Chinese Food and Drug Administration has approved salvianolic acids for the treatment of chronic angina [46].

Flavonoids, mainly represented by the glucuronides and the acyl glucuronides of luteolin, are the second class of polyphenols retrieved in all the wastewaters of this study. This is noteworthy, because the derivatives of luteolin, and in particular luteolin 7-*O*-glucuronide, have already demonstrated an interesting anticancer effect [47].

As concerns the identification of the compounds actually responsible for the antioxidant activity of the studied wastewaters, the limited amount of available data was certainly not enough to establish definite cause–effect relationships; however, several observations can be made about their general tendency.

Antioxidant power expressed as DPPH values gave the highest response in *T. vulgaris* and *O. vulgare*, whereas the measurements in terms of ORAC values stressed, besides these two species, the outstanding response of *S. officinalis*. Both multivariate and correlation analysis indicated an association between ORAC values and organic acid content. As a matter of fact, the decreasing ordered sequence of ORAC values (*S. officinalis* > *O. vulgare* > *T. vulgaris* > *O. majorana* > *R. officinalis*) recalls the ordered sequence for total organic acids content (*S. officinalis* > *T. vulgaris* > *O. vulgare* > *R. officinalis* > *O. majorana*). *T. vulgaris* and *S. officinalis* were the two species with the highest amounts of organic acids. Rosmarinic acid is an ester of caffeic acid (3,4-dihydroxycinnamic acid) and 3,4-dihydroxyphenyl lactic acid [40,48]. It was first isolated in (and named after) *R. officinalis*, but it is very common in many other species from Lamiaceae and Boraginaceae families, and many studies have stressed its large antioxidant potential, so much that it has been suggested in several medical applications [49,50]. Rosmarinic acid surely plays an important role in assessing the overall antioxidant capacity of the examined extracts. However, it may not be defined as the only substance responsible for the biological activity of the wastewaters, and other compounds cannot be ruled out. Caffeic acid and its many derivatives, for example, may have played a significant role as well.

None of the identified compounds could be claimed responsible for the antioxidant capacity of the wastewaters under study, and a reliable hypothesis is that all of them exert a synergistic action on the overall biological activity of the tested wastewaters. However, significant biological activity allows concluding that even these by-products of distillation can have potential for further use, and their exploitation could enhance the convenience and sustainability of the whole distillation process.

## 4. Materials and Methods

### 4.1. General

High-purity solvents were from VWR (Milan, Italy), reagents and reference standards were purchased from Sigma-Aldrich Products (Merck KGaA, Darmstadt, Germany), Extrasynthese (Lyon, France) and Fluka (Milan, Italy), and the biological material was from Amersham Bioscience (Little Chalfont, Buckinghamshire, UK), Cell Signaling Technology (Danvers, MA, USA) and Santa Cruz Biotechnology (Dallas, TX, USA).

### 4.2. Plant Material

The studied material was obtained from five MAPs belonging to the *Labiatae* family (*Rosmarinus officinalis* L., *Origanum vulgare* L., *Origanum majorana* L., *Salvia officinalis* L. and *Thymus vulgaris* L.) cultivated in Aragona (AG, Sicily, Italy), kindly provided by the company Rinoldo Davide srl, collected in the balsamic period in 2021 (typically May–June). The samples were identified by the Botany Department of the University of Catania, and specimens are kept in the herbarium of the Institute of Biomolecular Chemistry in Catania (voucher specimens: 001/2021, 002/2021, 003/2021, 004/2021, 005/2021, respectively).

### 4.3. Plant Hydrodistillation and Wastewater Recovery

Aerial parts of each species (100 g of air-dried material) were hydrodistillated by means of a Clavenger-type apparatus for about 3 h. After cooling, the essential oil and hydrolates were removed, and the wastewater was separated from the solid biomass through a filter paper filtration process (Whatman, cat. No. 1004−930, grade 4). Then, it was frozen and freeze-dried and stored at room temperature in sealed plastic Falcon tubes until analyses.

### 4.4. Hydrodistillation Wastewaters Analysis

A water solution (10 mg lyophilized wastewater/1 mL) for each plant species was filtered on PTFE 0.45 mm filters (PALL Corporation, Port Washington, NY, USA) and put into 2 mL amber vials for analysis. Polyphenols’ analysis was carried out on an Ultimate3000 instrument equipped with a photodiode array detector (Thermo Scientific, Rome, Italy). All chromatographic runs were performed using a reverse-phase column (Gemini C_18_, 250 × 4.6 mm, 5 μm, Phenomenex, Rome, Italy) equipped with a guard column (Gemini C_18_, 4 × 3.0 mm, 5 μm particle size, Phenomenex, Rome, Italy). Samples were eluted with a gradient of 5–90% buffer B (2.5% formic acid in acetonitrile) in buffer A (2.5% formic acid in water) over 50 min, after which the system was maintained for 7 min at 100% Buffer B. The solvent flow rate was 1 mL/min. Quantifications were carried out at 280 nm using gallic acid as a standard for phenolic acids (r^2^ = 0.999), at 330 nm using caffeic acid (r^2^ = 0.998), *p*-coumaric acid (r^2^ = 0.998) and ferulic acid (r^2^ = 0.999) as standards for cinnamic acids, and at 350 nm using apigenin 7 *O*-glucoside (r^2^ = 0.999) and luteolin 7 *O*-glucoside (r^2^ = 0.999) as standards for flavones and quercetin 3 *O*-rutinoside (r^2^ = 0.9987) as a standard for flavonols. To unambiguously identify the chromatographic signals and/or to confirm peak assignments, a series of HPLC/ESI-MS analyses was performed. The HPLC apparatus, solvent system and elution programs used were the same as described above, whilst ESI mass spectra were acquired as already reported in [4].

### 4.5. 2,2-Diphenyl-1-Picrylhydrazyl Free Radical (DPPH) Assay

The free radical scavenging ability of the samples (lyophilized wastewaters) was tested by the DPPH radical scavenging assay according to Chelly et al. [51]. Samples were solubilized in MeOH and 37.5 μL of the prepared concentrations (range: 0.125–1 mg/mL) were separately mixed with 1.5 mL of a DPPH methanolic solution (100 μM). Control tubes were prepared by adding an equal volume of MeOH (37.5 μL). Sample absorbance was recorded at 517 nm using a UV-visible spectrophotometer (Shimadzu, Kyoto, Japan) and after being incubated for 20 min at room temperature in the dark. A standard curve was prepared using the water-soluble analogue of vitamin E as a positive control (Trolox: 6-hydroxy-2,5,7,8-tetramethylchroman-2-carboxylic acid). Results were reported as µmol Trolox equivalents per gram of dry matter (dm) and expressed as mean ± SD of three different experiments carried out in triplicate.

### 4.6. Oxygen Radical Absorbance Capacity Assay

The oxygen radical absorbance capacity (ORAC) method was carried out according to Ouerghemmi et al. [52]. Briefly, an amount of each dilution of samples and Trolox (200 μL) were separately added to 117 nM fluorescein (1200 μL) and mixtures were stored for 15 min at 37 °C. After incubation, 600 μL of freshly prepared 40 mM AAPH (2,2′-azobis(2-amidino-propan) dihydrochloride) solution was added to the mixture. The fluorescence intensity (emission at 520 nm and excitation at 485 nm) was recorded during 90 min at 37 °C every 30 s by means of a Shimadzu RF-5301PC spectrofluorophotometer, and phosphate buffer was used as a blank (75 mM, pH 7.4). Experiments were performed in triplicate for each sample. The area under the curve (AUC) was determined as the difference between the blank (phosphate buffer) and samples (lyophilized water wastes) or the standard (Trolox) AUC. ORAC values were reported as µmol Trolox equivalents per gram of dry matter and expressed as mean ± SD of three different experiments carried out in triplicate.

### 4.7. Superoxide Anion Scavenging Activity

The superoxide dismutase (SOD) mimetic activity of the samples under study was measured by the reduction of nitro blue tetrazolium (NBT), as previously described by Abidi et al. [53]. Briefly, the non-enzymatic system phenazine methosulfate-nicotinamide adenine dinucleotide (PMS/NADH) generates superoxide radicals (O_2_^•−^), which reduces NBT to a purple formazan. Aliquots of 20 μL of the samples to be tested dissolved in MeOH at different concentrations or of the vehicle alone were added to 1.5 mL of a mixture containing 500 μL of NADH (73 μM), 500 μL of NBT (50 μM) and 500 μL of PMS (15 μM). All reagents were prepared in Tris/HCl (16 mM, pH 8). After incubation for 2 min at r.t., the absorbance was measured at 560 nm to determine the concentration of formazan formed. All experiments were carried out in duplicate and repeated at least three times. The results were expressed as percent decrease with respect to controls, and half maximal scavenging concentrations (SC_50_, mg dm/mL) and 95 % confidence limits (C.L. 95%) were calculated by using the Litchfield and Wilcoxon test.

### 4.8. TNF-α Induced Inflammation on Intestinal Epithelial Cells

#### 4.8.1. Cell Cultures

Caco-2 epithelial cells, obtained from ATCC, were grown in DMEM supplemented with 10% FBS, 4 mM L-glutamine, 1% non-essential amino acids, 100 U/mL penicillin, and 100 μg/mL streptomycin. Cells were maintained at 37 °C in a humidified atmosphere with 95% air and 5% CO_2_. To prepare Caco-2 monolayers, cells were plated at 4 × 10^4^ per cm^2^ on the upper side of transwell inserts (0.4 µm pore size; BD Falcon) and cultured for 18 days post-confluence to obtain fully differentiated cells [54]. Monolayer integrity and formation of tight junction were assessed by measurement of trans-epithelial electrical resistance (TEER) by using a Millicell-ERS Voltohmmeter (Millipore, MA, USA). Monolayers used in this study had TEER values ≥ 600 Ωx cm^2^. Differentiated Caco-2 monolayers, prepared as described above, were pretreated or not with the extracts (50 µg/mL) for 24 h, added only to the apical compartment of the transwell inserts. The extracts were always freshly dissolved in DMEM and immediately used. Dexamethasone (10 µM for 1 h) was used as a positive control. After 24 h, cells were washed twice with DPBS and then exposed for 6 h to tumor necrosis factor-α (TNF-α, 50 ng/mL) added in both the apical and the basolateral compartments of the transwell inserts. The TNF-α concentration was chosen on the basis of preliminary experiments indicating that exposure to 50 ng/mL significantly decreased the TEER value already after 3 h compared to the untreated control cells [54]. In all the experiments, cell monolayers incubated with the vehicles only were used as controls

#### 4.8.2. Whole Cell and Nuclear Lysates’ Extraction

Following the appropriate treatment, whole cell lysate was prepared in non-denaturing lysis buffer (10 mM Tris HCl, pH 7.4, 150 mM NaCl, 1% Triton X-100, and 5 mM EDTANa2) containing protease inhibitors (1 µg/mL leupeptin, 1 mM benzamidin, 2 µg/mL aprotinin) and 1 mM DTT. Nuclear extracts were prepared as described elsewhere [55]. The nuclear protein fractions were stored at −80 °C until use. Protein concentration in lysates was determined using the Bradford reagent [56] using bovine serum albumin (BSA) as a standard.

#### 4.8.3. Western Blot Analysis

For immunoblot analyses, 40 μg of protein lysates per sample were denatured in 4 × SDS-PAGE sample buffer (260 mM Tris–HCl, pH 8.0, 40% (*v*/*v*) glycerol, 9.2% (*w*/*v*) SDS, 0.04% bromophenol blue and 2-mercaptoethanol as a reducing agent) and subjected to SDS-PAGE on 10% acrylamide/bis-acrylamide gels. To determine the transcription factor NF-κB (nuclear factor kappa light-chain-enhancer of activated B cells) p65 nuclear level, nuclear lysates were used, and cyclooxygenase-2 (COX-2) levels were determined in whole cell lysates. Separated proteins were transferred to PVDF membrane (Hybond-P PVDF, Amersham Bioscience). Residual binding sites on the membrane were blocked by incubation in TBST (10 mM Tris, 100 mM NaCl, 0.1% Tween 20) with 5% (*w*/*v*) non-fat milk powder for 3 h at room temperature. Membranes were then probed with specific primary antibodies: rabbit anti-NF-κB p65 monoclonal antibody (Santa Cruz Biotechnology) (1:400), rabbit anti-COX-2 (Cell Signaling Technology, Danvers, MA, USA) (1:1000), mouse anti-β-Actin monoclonal antibody (Santa Cruz Biotechnology, Dallas, TX, USA) (1:700) and mouse anti-Lamin-B monoclonal antibody (Santa Cruz Biotechnology) (1:700), followed by a peroxidase-conjugated secondary antibody: anti-rabbit Ig (Cell Signaling Technology, Danvers, MA, USA) (1:5000) and anti-mouse Ig (Cell Signaling Technology) (1:5000), visualized with an ECL plus detection system (Amersham Biosciences, Little Chalfont, Buckinghamshire, UK). The equivalent loading of proteins in each well was confirmed by Ponceau staining and β-actin or Lamin-B control. Quantitative analysis was performed by densitometry.

### 4.9. Statistical Analysis

With the aim to identify any patterns of systematic variation of the obtained data, and to find relationships between the measured antioxidant activity of the examined wastes, all data were submitted to statistical analysis by means of the Minitab^®^ Statistical Software, version 17.1.0, and PAST 4.03 (State College, PA, USA) [57]. To meet the assumptions of the ANOVA, the Anderson–Darling test for normal distribution (as implemented in the Minitab software) was run, and when needed, non-normal data were subjected to Box-Cox transformation, according to the formula:

(1) $$Y^{\prime }=\frac{Y^{\lambda }-1}{\lambda }$$

where *Y*′ are the transformed data, *Y* are the original data, and λ may range between −5 and 5 [58,59]. In our dataset, the best normalization of data was obtained by assuming λ values of −3 (total compounds, flavones), −1 (ED_50_ values), 2 (flavonoids) and 3 (organic acids). The other variables did not need any transformation. A preliminary one-way ANOVA was therefore run on all transformed chemical data, setting the species as variability source, and differences among species were assessed by means of Tukey’s test [60].

To assess any significant trend in samples’ variability, the package PAST 4.03 was used to run correlation analysis and PCA. For all variables showing a significant correlation value, a regression analysis was conducted, and the relationship between the studied variables was observed by visual assessment by means of the software “Excel 365” for Microsoft Office Professional Plus 2016.

The data relative to the antioxidant/radical scavenger activity measured using the DPPH assay, the ORAC assay and the SOD-like activity assay, as well as data obtained from in vitro experiments on cultured cells, were statistically analyzed by a one-way or a two-way ANOVA, followed by Tukey’s HSD test, using the statistical software ezANOVA (https://people.cas.sc.edu/rorden/ezanova/index.html accessed on 4 April 2022). Differences among groups and treatments were considered significant at *p* < 0.05.

## 5. Conclusions

This study contributes to the knowledge on the polyphenolic composition of the distillation wastewater of four of the most widespread aromatic plants of the Lamiaceae family. Although further studies are necessary to complete the picture of the chemical composition of these residues by extending the analyses to the non-polyphenolic components of the matrix, our results highlight the presence of high quantities of compounds with proven biological activity (especially rosmarinic acid) and therefore of great interest in the pharmaceutical and nutraceutical fields. The exploitation of phytocomplexes based on these agro-industrial residues could have a positive effect both in environmental and economic terms of the aromatic plants’ supply chain.

## Figures and Tables

**Table 1:** Mean values (mg g^−1^ d.m.) of the major detected polyphenols in the wastewaters from five aromatic species, and results of the one-way ANOVA for each chemical group.

Compounds	Significance of F ^(1)^	*O. vulgare*	*T. vulgaris*	*S. officinalis*	*R. officinalis*	*O. majorana*
**Organic Acids Derivatives**	***	**94.08 ± 0.84 C**	**118.37± 0.87 B**	**123.38 ± 1.65 A**	**92.35 ± 0.55 C**	**88.22 ± 0.90 D**
Dihydroxyphenylacetic acid		20.39	2.79	6.28	17.31	4.46
Syringic acid		5.47	1.94	-	-	-
Benzoic acid derivative		1.36	1.45	-	-	2.49
Cinnamic acid derivative A		-	-	1.42	-	-
Feroulic acid derivative		3.68	6.04	3.88	4.63	2.23
Caffeic acid		-	0.36	-	-	0.52
Caffeic acid trimer A		-	-	1.60	-	-
Cinnamic acid derivative B		-	-	-	-	7.57
Syringoyl dihexoside		9.30	-	-	-	-
Salvianolic acid isomer A		4.55	-	-	-	-
Rosmarinic acid glucoside		-	3.05	-	-	1.51
Salvianolic acid isomer B		-	-	3.43	-	-
Caffeic acid tetramer A		-	-	13.85	-	-
Rosmarinic acid		49.35	66.37	54.78	70.41	53.98
Salvianolic acid K		-	18.43	35.92	-	-
Salvianolic acid isomer C		-	2.83	-	-	-
Caffeic acid trimer B		-	13.24	2.22	-	-
Caffeic acid tetramer B		-	-	-	-	15.46
Cinnamic acid derivative C		-	1.86	-	-	-
**Flavonoids Derivatives**	***	**30.77 ± 0.33 C**	**42.91 ± 2.51 B**	**51.88 ± 0.64 A**	**41.90 ± 0.07 B**	**41.53 ± 0.24 B**
**Flavones ^(2)^**	***	**30.77 ± 0.33 C**	**27.63 ± 2.50 C**	**42.74 ± 0.77 A**	**28.16 ± 0.02 C**	**39.93 ± 0.22 B**
Apigenin 6-8-di-*C*-glucoside		-	1.11	1.31	-	3.35
Luteolin derivative		6.55	-	-	-	-
Nepitrin		-	-	10.12	-	-
Apigenin derivative A		2.84	-	-	-	-
Luteolin 7-*O*-rutinoside		-	-	2.07	-	-
Luteolin 7-*O*-glucuronide		8.97	26.24	28.49	3.28	26.07
Apigenin X-*O*-glucuronide		-	-	-	-	10.51
Apigenin derivative B		5.50	-	-	-	-
Apigenin 7-*O*-glucoside		5.83	-	-	-	-
Luteolin 3-*O*-glucuronide		-	-	0.75	15.64	-
Luteolin X-*O*-acetylglucuronide A		-	-	-	3.35	-
Luteolin X-*O*-acetylglucuronide B		-	-	-	3.41	-
Luteolin X-*O*-acetylglucuronide C		-	-	-	2.47	-
Methyl apigenin X-*O*-glucuronide		1.08	-	-	-	-
**Flavonols**	***	**0.00 E**	**15.28 ± 0.15 A**	**9.14 ± 0.18 C**	**13.74 ± 0.07 B**	**1.60 ± 0.02 D**
Quercetin 3-*O*-glucoside		-	7.16	-	-	-
Quercetin derivative		-	3.65	-	-	-
Quercetin 3-*O*-rhamnoside		-	4.48	6.35	-	1.60
Quercetin 3-*O*-glucuronide		-	-	2.79	13.74	-
**Total**	***	**124.86 ± 1.17 E**	**161.28 ± 2.81 B**	**175.26 ± 1.46 A**	**134.24 ± 0.60 C**	**129.75 ± 1.12 D**

^(1)^ ***: means significantly different at *p* ≤ 0.001. Within each group, means not followed by the same letter are significantly different at *p* ≤ 0.05 (Tukey’s test). ^(2)^ The X in the names of some of the compounds reported below represents the uncertainty about the position of the sugar in the flavonoid skeleton.

**Table 2:** Antioxidant/radical scavenger activity measured using the DPPH assay, the ORAC assay and the SOD-like activity assay. All experiments were repeated three times.

	DPPH Assay μmol TE/mg dm	ORAC Assay μmol TE/mg dm	SOD-Like Activity Assay SC_50_ mg dm/mL (CL 95%)
*Origanum vulgare*	1.63 ± 0.13	4.40 ± 0.17 ^b^	0.967 (0.726–1.287)
*Thymus vulgaris*	1.79 ± 0.22	4.22 ± 0.06 ^b^	0.978 (0.738–1.295)
*Salvia officinalis*	1.49 ± 0.08	4.70 ± 0.11 ^b^	0.991 (0.747–1.315)
*Rosmarinus officinalis*	1.41 ± 0.15	3.35 ± 0.63	1.168 (0.856–1.592)
*Origanum majorana*	1.05 ± 0.22 ^c^	3.63 ± 0.26	2.102 ^a^ (1.658–3.029)

Results of DPPH and ORAC assays are expressed as mean ± SD. TE: Trolox equivalents; SC: scavenging concentration; CL: confidence limits; dm: dry matter. ^a^ *p* < 0.05 vs. all other extracts; ^b^ *p* < 0.05 vs. *Rosmarinus officinalis* and *Origanum majorana*; ^c^ *p* < 0.05 vs. *Origanum vulgare*, *Thymus vulgaris* and *Salvia officinalis.*

**Table 3:** Eigenvalues and percentage of explained variance for the 8 PCs found in the PCA.

PC	Eigenvalue	% Variance
1	3.93527	49.191
2	2.34933	29.367
3	1.43137	17.892
4	0.20556	2.5695
5	0.05069	0.6326
6	0.02786	0.3482
7	8.75278 × 10^−8^	1.0941 × 10^−6^
8	6.86664 × 10^−9^	8.5833 × 10^−8^

## Data Availability

The data that support the findings of this study are available upon reasonable request from the corresponding authors (E.N. and F.C.).
